# Radiotherapy in the Multidisciplinary Management of Adenomyoepithelioma of the Breast with an Axillary Lymph Node Metastasis: A Case Report and Review of the Literature

**DOI:** 10.7759/cureus.1380

**Published:** 2017-06-21

**Authors:** Natalie Logie, Judith Hugh, K Paulson, Robert Pearcey, Karen M King

**Affiliations:** 1 Cross Cancer Institute, University of Alberta; 2 Division of Anatomical Pathology, University of Alberta; 3 Radiation Oncology, University of Alberta; 4 Cross Cancer Center, University of Alberta; 5 Department of Medical Oncology, Cross Cancer Institute, University of Alberta

**Keywords:** adenomyoepithelioma, breast neoplasm, radiotherapy, lymph nodes, metastases

## Abstract

We describe a case of aggressive adenomyoepithelioma (AME) of the breast with a lymph node metastasis. A 63-year-old female presented with a fluctuating breast mass and clinically palpable lymph nodes. The patient underwent excisional biopsy followed by mastectomy with lymph node dissection and adjuvant radiotherapy (RT). Clinical behavior of both benign and malignant AME is described with the review of the literature and treatment recommendations.

## Introduction

Adenomyoepithelioma (AME) is a rare histologic subtype of breast tumors that comprise a spectrum of hyperplastic and neoplastic biphasic tumors composed of ductal or myoepithelial cells [1]. Clinically, AME of the breast presents in females over the age of 60 with a long standing history of a stable mass followed by a period of rapid growth [1]. AME of the breast has been associated with non-specific findings on mammography [2].

These tumors are typically described as benign in nature but, in some reviews have demonstrated malignant behavior [1,3-5]. As AME is a relatively rare pathologic entity with few case series. Here we review a case with a lymph node metastasis, discuss the literature, and provide the treatment recommendations. Informed consent statement was obtained for this study.

## Case presentation

### Presentation and imaging

A 63-year-old female presented with a right breast lump that was described as fluctuating in size over the month. She had a normal mammogram one year prior. She denied other changes in her breasts including nipple inversion or discharge, erythema or peau d’orange, or new masses in the axilla.

A mammogram was performed one month later demonstrating a well-defined lobulated opacity in the upper outer quadrant of the right breast. Ultrasound confirmed a 24 x 22 x 23 mm complex cyst at the 11 o'clock region of the right breast. In the right axilla, a prominent lymph node measuring 26 x 21 x 27 mm was visualized.

### Diagnosis and initial treatment

A needle-core biopsy was performed. This was non-diagnostic and so an excisional biopsy was performed. The excisional biopsy resulted in positive margins. The patient’s case was discussed at multidisciplinary tumor board and the consensus was reached to proceed to a right mastectomy with an axillary lymph node dissection. Surgery occurred two months after the excisional biopsy. A 3.5 cm tumor from the axilla was removed with an additional 25 axillary nodes excised.

### Pathological description

Needle core biopsies demonstrated chronically inflamed breast tissue with an infiltrative atypical squamous proliferation which was non-diagnostic. An excisional biopsy contained a 3 cm cystic cavity with both an intracystic and a 2.5 cm poorly marginated mural nodule that extended to the resection margin. The subsequent mastectomy and axillary lymph node dissection contained a small residual tumor focus in the breast and a 3.5 cm matted axillary nodal metastasis. Twenty-five other lymph nodes were negative for malignancy.

**Figure 1:**
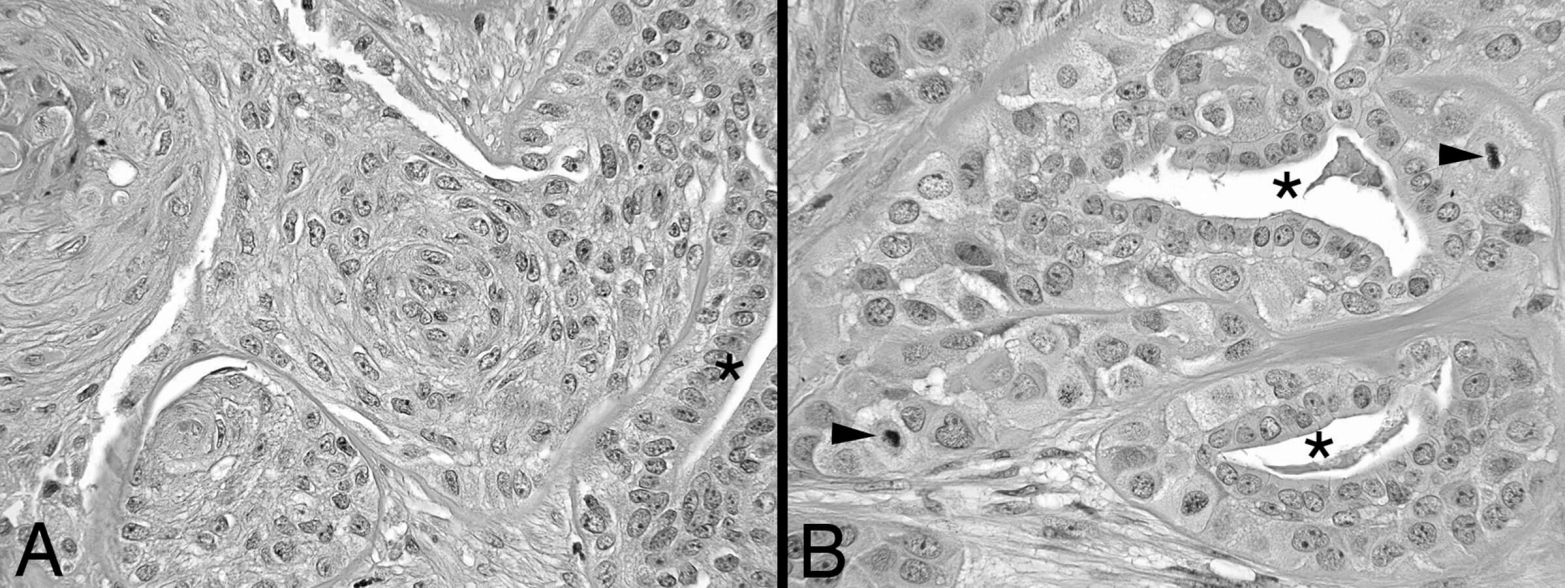
Hematoxylin and eosin stain showing a medium (X200) (A) and high (X400) (B) power view of the tumor. The biphasic composition of the tumor is demonstrated by the slit-like glandular spaces (asterisks) lined by benign cuboidal epithelial cells. The atypical myoepithelial cell component surrounds the luminal epithelium and shows squamoid differentiation (A) and mitoses (B) (shown by arrowheads)

The breast and axillary tumor appeared histologically identical. The tumor consisted of variably sized solid and infiltrating groups of cells within a desmoplastic stroma (Figure 1). There were numerous compressed glandular spaces lined by benign appearing cuboidal epithelial cells. These luminal cells were surrounded by polygonal myoepithelial cells with eosinophilic cytoplasm which rested on a well-demarcated basement membrane. The myoepithelial cells showed variable nuclear atypia and up to six mitotic figures per 10 high-power fields (MF/HPF). There was also the transition of the myoepithelial cells to spindle squamous cells with an intermediate or transitional type appearance with whorls and dyskeratotic foci. No myoid or cartilaginous differentiation was seen. The adjacent breast tissue showed a 7 mm focus of intermediate grade ductal carcinoma in situ and focal lobular carcinoma in situ, classic pattern. The case was referred to an external expert breast pathologist who made the diagnosis of an aggressive adenomyoepithelioma (infiltrative, lobulated and papillary types) with squamous differentiation based on the infiltrative pattern and mitotic activity. They noted that the presence of both cell types in the metastasis as seen in this case is rare but has been reported before [3].

### Radiotherapy (RT) description

After discussion at multidisciplinary tumor board, recommendations were made for adjuvant RT to the chest wall and lymph nodes without adjuvant chemotherapy or endocrine therapy. Two months after her mastectomy and lymph node dissection, the patient was treated with 45 Gray (Gy) in 20 fractions using a four-field photon RT technique. A tangent pair (with a field-in-field plan to improve dose homogeneity) was used to treat the chest wall, lower axilla, and internal mammary chain lymph nodes. An anterior/posterior pair was used to treat the supraclavicular lymph nodes and upper axilla. Three years after completion of RT, there was no evidence of recurrent disease.

## Discussion

The defining characteristic of AME lesions is the presence of both the glandular and myoepithelial elements. These are part of a spectrum of myoepithelial lesions which include myoepitheliosis, adenomyoepithelial adenosis, and AME lesions. There are three variants (spindle, tubule, and lobulated) [3]. Tavassoli, et al. suggested that patients with tubular variants are more likely to recur despite a non-aggressive morphology due to ill-defined boundaries with pathologic tubules blending with normal ducts [3]. AME can be either benign or malignant change with the separation based both on the cytologic features as well as the frequency of mitotic figures. Benign lesions generally have mitotic activity in the range of ≤ 2 MF/10 HPF) [6]. Malignant AME is a distinct entity in which either epithelial or myoepithelial components become malignant, giving rise to carcinoma [6].

The literature review has described cases of malignant AME with metastasis occurring in approximately 26%-40% of patients with metastasis to lung, bone, brain, liver, skin, and kidney reported [1,3-5]. Metastasis has also been reported in patients with benign AME (one case with a lymph node metastasis and the second case with chest wall recurrence followed by lung and brain metastasis) [3-4]. While not meeting criteria to be classified as malignant AME, both of these tumors with metastasis demonstrated 8MF/10 HPF. The case presented here demonstrates an aggressive AME with up to 6MF/10 HPF.

The benign and malignant AME tend to recur locally [4]. Recurrences have been reported with the median time to recurrence of 2.3 years [0.7- 5.7 years] (Table 1). The majority of recurrences reported have been in patients with excisional biopsy alone [3-4,7]. Management of these tumors with complete excision has been supported in the literature with case series showing no documented recurrences using this strategy [8]. It has been reported that the best predictor of recurrence is incomplete excision [4]. There is additional uncertainty for patients with incomplete excision due to a potential for malignant transformation [2]. For the patient reported here, mastectomy followed the excisional biopsy due to the increased risk of recurrence after pathologically positive margins.

**Table 1:** Case series of adenomyoepithelioma of the breast including patients with recurrence and outcomes N/A= not availabe yrs= years n= number of patients

Study	Initial treatment (n)	Patients with recurrence	Pathology	Time to recurrence	Follow-up treatment
Rosen, *et al.* (n=18)	Mastectomy (1) Complete excision (2) Excisional biopsy (15)	Two patients (all after excisional biopsy)	N/A	2.8 yrs	Re-excision
			N/A	1yrs	Re-excision
Tavassoli, *et al. *(n=27)	Mastectomy (9) Excisional biopsy (18)	Three patients (all after excisional biopsy)	Lobulated	0.7 yrs	Mastectomy
			Tubular	5.3 yrs	Mastectomy
				2.3 yrs	Re-excision
Loose, *et al.* (n=6)	Mastectomy (1) Partial mastectomy (1) Excisional biopsy (4)	Three patients (all after excisional biopsy)	Tubular	4 yrs	Re-excision
			Lobulated	5.7 yrs	Re-excision
			Lobulated	1.3 yrs	Mastectomy
McLaren, *et al.* (n=23)	Mastectomy (N/A) Complete excision (N/A)	None			
Moritz, et al. (n=14)	Mastectomy (4) Partial mastectomy (3) Excisional biopsy (2) Core biopsy (5)	None			

Axillary node dissection is not indicated for patients with AME except in the case of clinically involved nodes [1]. Lymph node metastasis has been rarely documented for benign AME. In a previous report of AME with a lymph node metastasis, the tumor featured both high MF/HPF and may have been direct extension to the nodal basin [3]. Here, we also report a tumor with high MF/HPF and a primary tumor location in the axillary tail with proximity to the lymph node conglomerate.

There have been few cases of either RT or chemotherapy utilized for AME [1,9]. One case report describes chemotherapeutic response for metastatic disease using eribulin [9]. Another case report described recurrent AME (a fourth recurrence after excisional biopsy, repeat excisional biopsy, and mastectomy) treated with 60 Gy RT to the chest wall, resulting in 15 months of local control but then later progressing to develop lung metastasis [4]. Case reports of malignant AME have utilized RT (50 Gy in 25 fractions followed by a 9 Gy in three fraction boost) where, despite mastectomy, there was tumor progression [10]. Adjuvant RT was employed in our patient to achieve local control in the setting of AME with a lymph nodes metastasis after resection.

A link between upregulation of ATM protein in malignant AME and possible radioresistance has been reported [10]. Proponents of RT have advocated that if RT is to be employed, minimal postoperative delays, higher doses, and accelerated fractionation should be considered [10]. In the case report here, RT was delivered at the standard post-mastectomy dose for our institution and has achieved disease control at the three-year follow-up period.

## Conclusions

Adenomyoepithelioma (AME) is a rare pathological entity of the breast. We advocate for management strategies providing local control for these patients including complete excision or partial mastectomy. Adjuvant RT may provide additional local control in the setting of AME with axillary metastasis. Recurrences are seen up to five to six years post therapy and close follow-up is required. There may be a link between radioresistance and malignant AME and modification of RT strategies (including accelerated RT and higher doses) may be required if RT is utilized for definitive purposes rather than adjuvant purposes.
